# Fearless in Physical Activity: The Implications of Community-Based Physical Activity Interventions on Children, Adolescents, and Adults with Congenital Heart Disease

**DOI:** 10.3390/jcdd10010011

**Published:** 2022-12-28

**Authors:** Adam Chubbs-Payne, Jenna Yaraskavitch, Lillian Lai, Jennifer Graham, Poppy DesClouds, Patricia E. Longmuir

**Affiliations:** 1 Children’s Hospital of Eastern Ontario Research Institute, Faculty of Medicine, University of Ottawa, Ottawa, ON K1H 8L1, Canada; 2 Children’s Hospital of Eastern Ontario Research Institute, Ottawa, ON K1H 8L1, Canada; 3 Children’s Hospital of Eastern Ontario, Ottawa, ON K1H 8L1, Canada; 4 Canadian Congenital Heart Alliance, Toronto, ON M4N 3P6, Canada; 5 Faculty of Health Sciences, University of Ottawa, Ottawa, ON K1N 6N5, Canada; 6 Children’s Hospital of Eastern Ontario Research Institute, Faculty of Medicine and Faculty of Health Sciences, University of Ottawa, Ottawa, ON K1H 8L1, Canada

**Keywords:** physical activity, children, adolescents, adults, congenital heart disease, recreation

## Abstract

People living with CHD do less moderate-to-vigorous activity than their peers. This study sought to examine the impact of a community-based physical activity intervention for individuals with CHD. Individuals with CHD and family members participated in a 3 h, one-day Fearless event consisting of a variety of physical activity and education sessions. Consenting participants completed self-administered questionnaires pre-/post-event and completed a post-event feedback form. Descriptive statistics and paired *t*-tests were calculated across subgroups for each outcome/questionnaire. Written feedback was analyzed using a six-phase framework of reflexive thematic analysis. A total of 32 participants (six children, six adolescents, five youth, five all ages, and ten adults) with CHD completed this study. Following Fearless, youth with CHD reported spending less time being ‘inactive’ and more time being ‘somewhat active’. Adults with CHD reported spending more time walking and partaking in moderate activity and less time partaking in vigorous activity. Fearless successfully engaged individuals with CHD who were more sedentary, less active, and older. Fearless is a fun, family-friendly, physical activity intervention for individuals with CHD. Attending a Fearless event helped children, adolescents, and adults with CHD make incremental improvements to their physical activity levels and provided a framework for sport and recreation leaders who aim to promote physical activity amongst individuals with CHD.

## 1. Introduction

Congenital heart disease (CHD) is the most common congenital condition and individuals with CHD comprise a large and rapidly growing population; approximately 2% of children are born with a CHD [1]. Recent developments in cardiovascular medicine and surgery have led to significant improvements in the life expectancy and quality of life of individuals with CHD, allowing 97% of these individuals to reach adulthood [2,3]. Early and regular access to physical activity as a child is key to establishing life-long healthy behaviours. It is known that physical activity improves the overall health of individuals with CHD, helping to reduce their elevated baseline cardiovascular risk [4,5]. It has been reported that 4% of girls and 12% of boys with CHD perform the daily physical activity recommended for optimal health [6]. These proportions are nearly identical to data for healthy girls (4%) and boys (9%) [7] evaluated using the same methodology. However, these results are concerning given the very small proportion of children who are sufficiently active for optimal health [8]. Furthermore, the average minutes for these populations as a whole are quite different. Boys (49 min/day) and girls (39 min/day) with CHD [6] performed only 80% of the daily physical activity of their healthy peers (boys 61 min/day; girls 47 min/day) [7]. These differences are similar even when the CHD is mild [9,10]. Moreover, few individuals with CHD have such severe disease as to require significant physical activity restrictions; therefore, both children and adults with CHD are encouraged to abide by public health physical activity recommendations and physical activity should be addressed and encouraged at every clinical encounter [10,11,12].

Despite recommendations, only 61% of adolescents and 19% of adults with CHD report receiving health care provider advice encouraging activity or conveying physical activity recommendations [13,14]. It is concerning that 18% of adolescents and 30% of adults with CHD report being discouraged or warned about physical activity [13,14]. Uncertainty and misconceptions surrounding physical activity safety and current recommendations (e.g., activity type and intensity level), and a lack of self-efficacy and confidence for activity participation are important barriers [4,11,15,16,17]. Questions such as “How much is too much?” and “Will it be too much for my heart?” are top of mind. Numerous calls have been made for the development and implementation of physical activity programs for individuals with CHD to help promote physical activity amongst this population [9,11,18]. Despite this, interventions completed to date have focused on exercise to improve physical fitness [19,20] rather than targeting daily physical activity participation.

Fearless Physical Activity (Fearless) events were created to promote safe and health-appropriate community-based physical activity for individuals with CHD. Fearless events were designed to be fun and to enhance physical activity confidence—providing children, adolescents, and adults living with CHD the opportunity to do physical activity “without fear” (i.e., worrying about health-related limitations). Fearless thus provides a framework for sport and recreation leaders who wish to promote physical activity amongst individuals with CHD. This study sought to: (1) determine the perceptions of individuals with CHD towards physical activity and Fearless events, (2) evaluate the impact of Fearless on intrinsic motivation, perceived competence, and current physical activity behaviour, and (3) utilize participant perceptions and outcomes to inform best practices for community-based physical activity opportunities for those with CHD. We hypothesized that Fearless event participation would increase positive perceptions of physical activity.

## 2. Results

### 2.1. Participant Demographics

A total of 322 individuals (including those with CHD and their family members) responded to participation recruitment efforts. Of these, 274 participants (117 individuals with CHD, 157 family members; 38 adults, 36 youth (adolescents and children), 88 children, 59 adolescents 53 all age registrants) took part in the 31 Fearless events. Of the 117 individuals with CHD who attended Fearless events, 32 (27%; six children, six adolescents, five youth, five all ages, and ten adults) consented to research participation. A subset of participants completed each questionnaire with a maximum of 16 youth and 16 adults completing a single questionnaire. Youth participants ranged from five-to-fifteen years of age (8.88 ± 3.61) while adult participants ranged from 26-to-54 years of age (40.56 ± 9.91; see Table 1). The youth pre-event questionnaires were completed within two months prior to the event $\bar{x}$ = seven days) and the post-event questionnaires were completed up to one month and 24 days post-event ($\bar{x}$ = one month and 18 days). The adult pre-event questionnaires were completed up to 19 days prior to the event ($\bar{x}$ = six days) and the post-event questionnaires were completed up to two months post-event ($\bar{x}$ = one month and nine days). Of the 274 event participants, 125 participants (78 youth, 25 adults, 22 attendees at all age event) completed the written feedback form.

### 2.2. The Intrinsic Motivation Inventory

There was no significant difference and a small effect size (Table 2) in intrinsic motivation pre- versus post-event or between those who completed only the pre-event questionnaire and those who completed both the pre- and post-event questionnaires. Youth who completed only the pre-event questionnaire (7.50 ± 3.16) were younger (*p* = 0.07, Cohen’s d = 0.82) than those who completed both the pre- and post-event questionnaire (10.25 ± 3.50). Adults did not differ by age (*p* = 0.40, Cohen’s d = 0.45).

### 2.3. Perceived Competence Scale

There were no significant differences and only small effect sizes (Table 2) in participants’ perceived competence from pre- to post-event. Perceived competence was higher among youth who only completed the pre-event questionnaire compared to those who completed the questionnaires pre- and post-event, with a large effect size. Adult perceived competence did not vary with questionnaire completion. Youth who completed the pre-event questionnaire only (7.50 ± 3.16) were younger (*p* = 0.13, Cohen’s d = 0.80) than those who did both the pre- and post-event questionnaires (10.25 ± 3.69). Adults did not differ by age in their questionnaire completion.

### 2.4. Habitual Activity Estimation Scale Total Physical Activity Levels

**Figure 1:**
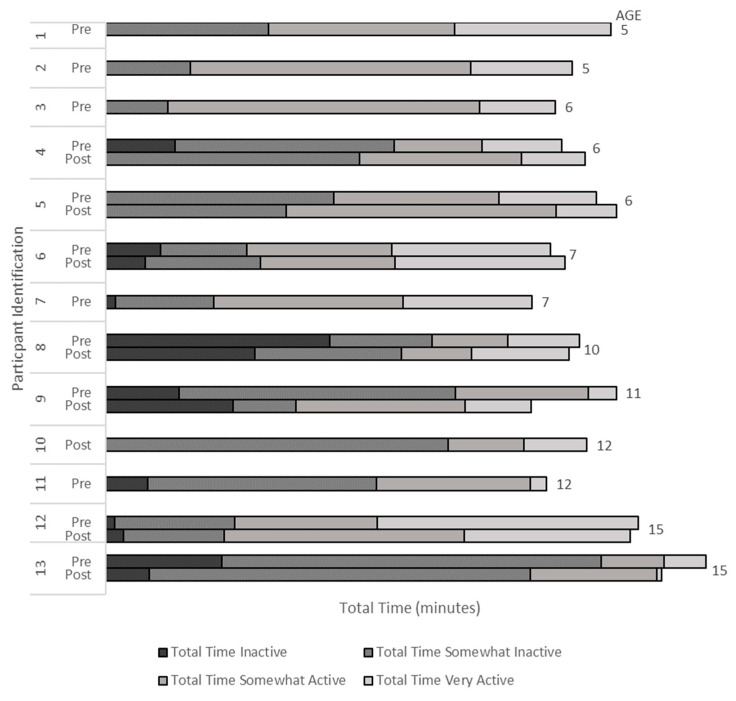
The 7-Day Physical Activity in Total Time (minutes) Reported by Youth in the Habitual Activity Estimation Scale.

Complete weekday and weekend Habitual Activity Estimation Scale responses were from thirteen youth (six pre-event only (three men); seven pre- and post-event (four men); Figure 1). Participants who completed both the pre- and post-event questionaries (n = 7) tended to have fewer minutes considered ‘inactive’ (*p* = 0.25, Cohen’s d = 0.35) with significantly more time reported as ‘somewhat active’ (*p* = 0.026, Cohen’s d = 0.92) post-event. No differences were found in the ‘somewhat inactive’ time (*p* = 0.499, Cohen’s d = 0.23) or the ‘active’ time (*p* = 0.40, Cohen’s d = 0.22). Participants (n = 7) who completed both pre- and post-event questionnaires tended to have more minutes of ‘inactive’ time (*p* = 0.052, Cohen’s d = 1.27) and less time considered ‘somewhat active’ (*p* = 0.066, Cohen’s d = 1.25) compared to those who completed only the pre-event questionnaire (n = 6). Participants who completed both the pre- and post-event questionnaires were not significantly older (*p* = 0.30, Cohen’s d = 0.60) than those who completed the pre-event questionnaire only. Youth reported they were active (4.2/6.0; 1.0 = very inactive, 6.0 = very active) and that the data collected were somewhat representative of a typical day (1.75/4.0; 1.0 = not very representative, 4.0 = very representative).

### 2.5. The International Physical Activity Questionnaire

**Figure 2:**
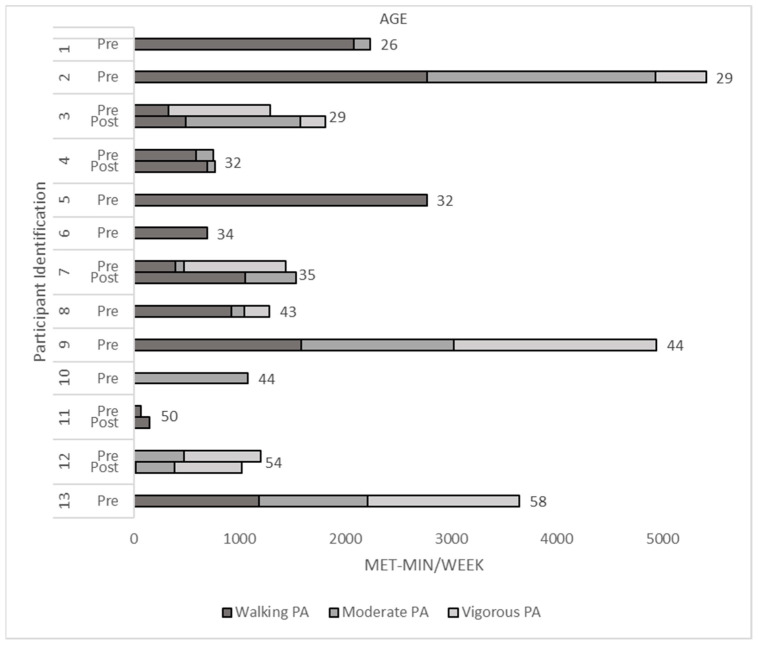
The 7-Day Physical Activity in MET-min/week of Adults Reported by the International Physical Activity Questionnaire.

Thirteen adults with CHD (eight pre-event only (1 man); five pre- and post-event (0 men) completed the International Physical Activity Questionnaire. Participants who completed both the pre- and post-event questionnaire (n = 5) reported an improvement in walking (*p* = 0.37, Cohen’s d = 0.60) and moderate-intensity activity (*p* = 0.26, Cohen’s d = 0.77) as well as a reduction in vigorous activity after attending a Fearless event. There was no change in total physical activity (*p* = 0.78, Cohen’s d = 0.18; Figure 2). The amount of walking (MET-min/week) was higher among adults who completed only the pre-event questionnaire (n = 13, *p* = 0.012, Cohen’s d = 1.64) compared to those who completed both the pre- and post-event questionnaires. Participants (n = 13) who completed the pre-event questionnaire only reported somewhat more moderate (*p* = 0.16, Cohen’s d = 0.78), and vigorous activity (*p* = 0.48, Cohen’s d = 0.37), and significantly more total (*p* = 0.057, Cohen’s d = 1.13) physical activity when compared to those who completed both questionnaires. These participants (36.75 ± 7.34) tended to be younger (*p* = 0.19, Cohen’s d = 0.67) than those who did both the pre- and post-event questionnaire (43.1 ± 11.4).

### 2.6. Post-Event Feedback

Three main themes emerged from the participants’ feedback (details in Supplementary Files S2b and S2c) relating to the ways in which Fearless is beneficial for individuals with CHD and their families: (1) Fearless is fun and inclusive, (2) Fearless helps participants establish healthy behaviours, (3) Fearless helps foster a sense of community and connection. The majority of participants found that Fearless events were a positive experience for a wide variety of individuals with CHD and their families. One participant stated, “It’s a lot of fun for the whole family. The fact that the kids have heart conditions is almost incidental–everyone seemed to be having a great time!” Participants viewed Fearless as integral to establishing healthy behaviours and fostering a sense of community and connection among peers with CHD and their families. One participant reported, “Living with CHD does not mean living without activity. The best gift we can give our daughter is the chance to play and move like her heart-healthy friends.” Moreover, another participant said “I enjoyed the afternoon. It was a great way to meet people and to learn about the breadth of activities we should consider in our everyday lives to improve our overall fitness and health.” Recommendations for improving the Fearless program included 1. having more families attend each event, 2. separation of the events by age (i.e., children, adolescents, and adult), 3. integrating a wider variety of activities (i.e., pilates, relay race, and yoga), and 4. logistical suggestions (i.e., improve efficiency and provide adequate time).

## 3. Discussion

This study sought to evaluate the impact of the Fearless community-based physical activity intervention on physical activity perceptions and participation among youth and adults with CHD. Results from the relatively small (27%) number of Fearless participants who consented to research participation suggest that Fearless supported improvements in physical activity behaviour and reduced sedentary time, with larger changes occurring among inactive individuals. Fearless events were perceived as positive, fun, family-friendly experiences that were beneficial for individuals with CHD and their families.

The 27% (32 of 117 individuals with CHD) of Fearless participants who consented to research does not allow these results to be generalized to all Fearless event participants or the broader CHD population. Whether Fearless participants who chose not to consent to research were different from study participants is unknown. However, previous research in this population suggests that adults with CHD are more likely to volunteer for research if their disease is more severe [21]. Our results also indicated that participants who completed both pre- and post-event questionnaires tended to be less active and older compared to those who only completed the pre-event questionnaires. These considerations are encouraging as they suggest that Fearless events may differentially target those most needing support for a healthy, active lifestyle.

### 3.1. Fearless Impacts on Physical Activity Motivation and Perceived Competence

Perceived physical activity competence and reported activity tended to be higher among youth completing only pre-event questionnaires. The relationship between physical activity level and self-concept/perceived competence in both children and adults is well-known [22], including among those with CHD [23,24]. Youth with higher activity and competence may have been less invested in, or influenced by the Fearless project that targeted inactive individuals leading to lower post-intervention compliance. Children who completed only the pre-event questionnaires also tended to be younger. As children age, their cognitive development shifts towards becoming more formal and concrete [25]. As such, younger children are more likely to be confident in their abilities as they have not begun to compare themselves to peers [25].

The relatively small study sample and the very small scale of the Fearless intervention (i.e., one half-day session) may have limited the ability to identify impacts via standardized questionnaires. Behaviour change theory [26] suggests that physical activity participation and psychological variables (i.e., competence and motivation) are linked in a bi-directional relationship among healthy children and adolescents [22]. The measurable impact of the Fearless intervention may have been greater if, aligned with participants’ feedback, sessions were offered on a weekly basis.

### 3.2. Fearless Impacts on Physical Activity Behaviour

Fearless events were focused on promoting the benefits of light-to-moderate activity among individuals with CHD. Self-reported physical activity behaviour improved following participation in just one 3 h Fearless event. Following Fearless, youth with CHD reported spending less time being ‘inactive’ and more time being ‘somewhat active’. Adults with CHD reported a shift in activity architecture, whereby they spent more time walking and doing activity they reported as moderate while also reporting less vigorous activity. The behaviour changes reported by both youth and adults with CHD reflect a positive impact of the Fearless activity promotion program. The small research sample size made it difficult to appreciate significant differences, but the effect sizes suggest that significant benefits may be seen in a larger sample. These incremental changes (i.e., inactive to somewhat inactive) were expected given the short intervention duration and the time requirements for effective behaviour change [26]. Most exercise training programs for those with CHD are 12 weeks in duration with three sessions per week and that at least nine weeks was required for long-term sustainability [20]. All Fearless sessions included discussion of physical activity recommendations for individuals with CHD. Participants’ reported shifts in activity behaviour may reflect a new understanding and more all-encompassing view of physical activity (i.e., reclassifying vigorous to moderate).

While a small proportion of youth with CHD achieve recommended physical activity levels, individuals with CHD who do not perform the recommended physical activity are less active than their heart-healthy peers [6,27]. As such, providing physical activity education and assisting in the establishment of healthy physical activity behaviours in individuals with CHD is imperative to their overall physical and mental health. As with their heart-healthy peers, physical activity decreases and sedentary time increases from childhood to adolescence among individuals with CHD [6,28]. Inactivity amongst adolescents with CHD is associated with more sedentary behaviours in adulthood and ultimately a larger burden of disease [13]. CHD-related health issues that impact physical activity also increase with age [29] and there is an inverse relationship between physical activity participation and quality of life [30]. Results from this study align with the small improvements in physical activity behaviour previously reported after interventions with children, adolescents, and adults with CHD [19]. One day of motivational interviewing followed by individualized home-training plans increased daily moderate-to-vigorous physical activity among adolescents with CHD [31]. Simple physical activity interventions (i.e., walking) are feasible, safe and improve the physical activity of adults with CHD [4].

Engaging sedentary individuals in physical activity interventions is difficult, even though these individuals would benefit most from participation [32]. Behaviour modification is known to be particularly difficult among adults with CHD [33]. Fearless was able to successfully engage more sedentary, less active, and older individuals, which is a promising first step in mitigating the sedentary lifestyle health risks of these individuals [12]. Results from this study suggest that future research should investigate the impact of an expanded Fearless intervention, focused on enhancing light-to-moderate physical activity and reducing sedentary behaviour rather than fitness training. Such changes are practical and sustainable physical activity behaviour modifications [11,12]. Offering Fearless events on a more frequent and regular basis may enhance the positive trends for changes in physical activity motivation and behaviour observed in this preliminary study, ultimately improving the mental and physical health in this population [13].

### 3.3. Perceptions of the Fearless Physical Activity Intervention

Fearless events were one-day, three-hour events held each season of the year in locations across Ontario, Canada for individuals with CHD of all ages and their families. One event per location per season was planned to reduce participation burden, allowing individuals to select the number of events attended, and offer activity options for all times of the year. Participants reported that Fearless was fun, family friendly, and for everyone. Fearless events were felt to improve the health behaviours of individuals with CHD and foster a sense of community. These findings align with recommendations that children with CHD should participate in a wide variety of developmentally appropriate physical activities [11] that focus on fun [34] and enjoyment [11]. Adults tend to be more motivated by increased responsibility for managing their own health [34]. Feedback from participants emphasized the desire for more frequent events in order to build physical activity habits with Fearless support.

Participants viewed Fearless as integral to the promotion of physical activity and the establishment of healthy physical activity behaviours among individuals with CHD. They emphasized that a strength of Fearless was its ability to benefit not only individuals with CHD but also their families. A family-based approach builds knowledge and awareness among patients and their families, and community programs increase opportunities [10]. The involvement of families helps children with CHD to adopt a positive physical activity perspective and establish behaviours that are carried into adulthood [34]. The inclusion of non-traditional activities in the Fearless events has previously been identified as critical to successful physical activity promotion among individuals with CHD [35]. While most participants found that Fearless was well designed, provided a wide variety of activities, and was an overall positive experience for those involved, the main recommendations for improving the program were to expand outreach to increase participant involvement and logistical suggestions such as further separating the events by age and integrating a greater variety of activities into the program.

### 3.4. Strengths and Limitations

Strengths of the current study include its diverse sample of participants with CHD with respect to age and baseline physical activity level, enabling consideration of these factors in the analyses of physical activity motivation and behaviour, as well as participants’ perceptions of the intervention. Qualitative data were coded independently by two researchers, which encouraged open discussion and helped mitigate bias [36]. A unique aspect of this study was the inclusion of family members in the Fearless intervention, reinforcing the importance of community and support for successful behaviour change.

It is also important to acknowledge the potential limitations of this study when interpreting the findings. Participating in this study required the individual with CHD to self-register for the event. Despite recruitment efforts, only 32 of 117 Fearless participants with CHD consented to research participation. The implications of the small sample size were offset by the diversity of participants and the use of valid and reliable outcomes. Research participants who did and did not complete both questionnaires differed with regard to level of activity and age, a difference considered during data analyses. Fearless participants who declined research participation were similar to those who consented with regard to age and sex. Social desirability bias was limited by enabling anonymous responses via an online platform. Data on patient diagnoses or disease severity were not collected. It is recognized that subjective measures of physical activity likely overestimated actual behaviour, particularly among children [37,38], but this study examined only changes over time with each person compared to him/herself. To optimize accuracy, parents were asked to involve their children in questionnaire completion but this was not verified. Youth indicated that their responses regarding physical activity levels were only ‘somewhat representative’ of a typical day.

## 4. Materials and Methods

### 4.1. Participants and Recruitment

Eligible participants for the Fearless events were children, adolescents, and adults with CHD (diagnosed by their most responsible physician), as well as family members of these individuals. The Canadian Congenital Heart Alliance (CCHA), a national patient and family support network, contributed to participant recruitment via the promotion of Fearless events to their members and the public using their website, social media platforms, existing patient networks, and cardiac care clinics throughout Ontario, Canada. Passive recruitment was done via a message on the Fearless registration page of the CCHA web site. Additionally, paediatric and adult congenital heart disease clinics across Ontario, Canada disseminated information about the Fearless events on behalf of the CCHA to their respective patients.

### 4.2. Data Collection

#### 4.2.1. Events

The CCHA offered a series of one-day Fearless events for individuals with CHD and their families (see Supplementary File S1 for examples of event design and content). Events were three hours in duration and included individuals of all ages. Events were either targeted for a specific age group (i.e., children, adolescents, and adults) or were combined (i.e., children and adolescents, all ages) to ensure an adequate number of participants for each session. For this paper, the term youth will be used to describe the combination of both children and adolescents together. A total of 31 Fearless events were hosted by community partners, including the MLSE Launch Pad (Toronto), Variety Village (Toronto), the Canada Games Complex (Thunder Bay) and YMCA/YWCA facilities (Ottawa, Sudbury, London). These community partnerships were designed to allow Fearless to be sustainable beyond completion of the research study. Events took place in each season of the year, within four regions of Ontario, Canada. Fearless events combined educational sessions (e.g., open brainstorm sessions, creative activities, and group discussions) with a wide variety of community physical activity opportunities (e.g., geocaching, frisbee golf, and croquet; Table 3). Although activities changed over the seasons and events, the structure of the event remained consistent including an introduction, an icebreaker, alternating physical activity opportunities and education sessions, and a discussion/feedback session. Where appropriate, the education topics were integrated into the physical activity opportunities (e.g., for self-monitoring, participants checked in with how their body was feeling during an activity). Activities were chosen based on participants’ age, the interests and needs of individuals with CHD, and options available in local communities at little to no cost (e.g., use of local trails and parks). It is important to note that one event was cancelled due to a low number of participants.

#### 4.2.2. Recruitment

All Fearless participants were asked to partake in this research study but were informed that attendance at these events was not contingent on study participation. Participants who consented to research participation were contacted via email to provide access to RedCAP, an online platform where participants completed self-administered questionnaires. All data were stored electronically in a secure password-protected and encrypted location. No identifiable information was collected in the RedCAP surveys or feedback forms. For this study, participants’ implied consent was obtained through their selection of the links to the research information and voluntary completion of the questionnaires. All participants, both individuals with CHD and their families, were asked to complete a feedback form at the end of each Fearless event.

#### 4.2.3. Assessments and Outcomes

Participants’ physical activity level and activity motivation were measured both pre- and post-event using standardized self-report questionnaires distributed via RedCAP. All consenting participants were sent the pre-event questionnaire two weeks prior to the event or at the time of registration (if within two weeks of event). The post-event surveys were sent to participants two weeks following the event and all post-event surveys were completed within eight weeks. Some participants were lost to follow-up resulting in the completion of only the pre-event questionnaire instead of both the pre- and post-event questionnaires.

Intrinsic Motivation Inventory (IMI). This multidimensional questionnaire, for both youth and adults, measures individuals’ subjective experience while participating in an activity or task [39]. A subset of the IMI questions, focusing on the subscales of interest/enjoyment and pressure/tension, were selected, and modified to specify “physical activity” as the activity of interest at the Fearless event. Participants rated each statement on a scale ranging from 1, not at all true, to 7, very true. An increase in enjoyment/interest and/or a reduction in pressure/tension for physical activity were hypothesized after the Fearless intervention.

The Perceived Competence Scale (PCS). This brief 4-item questionnaire for is used to measure self-perceived competence in both youth and adults for a certain task. For this study the task was defined as physical activity. Participants rated each statement on a scale ranging from 1, not at all true, to 7, very true. Self-perceived competence, which is foundational for activity participation, was hypothesized to increase after the Fearless intervention.

The Habitual Activity Estimation Scale (HAES). This questionnaire was administered to youth participants to assess their daily physical activity [40]. Participants were asked to recall the activities of a “typical” day during the week and weekend. Days were divided into four time periods (bed to breakfast, breakfast to lunch, lunch to supper, supper to bed). Participants were asked to estimate the percentage of time spent doing each of the four different activity intensities (inactive, somewhat inactive, somewhat active, very active) during each of the four time periods. (e.g., 60 min inactive from bed to breakfast). Researchers asked two additional questions “how would you describe your overall level of activity” (“very inactive” to “very active”) and “how representative was this of a ‘typical’ day” (“a little like most days” to “a lot like most days”).

The International Physical Activity Questionnaire (IPAQ). The shortened version of this questionnaire was administered to adult participants, to assess their daily physical activity [41]. This questionnaire considered a wide variety of physical activities and required participants to provide the number of days per week and total time per day (minutes) spent doing a certain intensity of activity (sedentary, walking, moderate, and vigorous). The IPAQ has been shown to be a quick and reliable indicator of physical activity amongst adults with CHD [30].

Post-Event Feedback. Participants with CHD and their family members were asked to complete a written feedback form at the end of each event (Supplementary File S2a) with prompts such as, “share a short testimonial or quote about your experience” and “provide comments or suggestions about how we could make this event more helpful”. Consenting individuals completed the post-event feedback form, summarizing their experiences and providing suggestions for how to improve future physical activity interventions.

### 4.3. Data Analysis

Data from the IMI, the PCS, the HAES, and the IPAQ were collected, and reverse scoring was conducted when necessary (e.g., IMI). Frequency tabulations described categorical variables such as subgroups (all participants pre-event; participants who completed the pre-event questionnaire only; participants who completed both pre- and post-event questionnaires); age group (youth, adult) and perceived gender for each of the five questionnaires. For this study, children and adolescents were combined to form one group (youth) given that children and adolescents were occasionally combined during Fearless events to ensure an adequate number of participants for each session. Descriptive statistics (mean + standard deviation) were calculated across subgroups for each of the five questionnaires. Paired *t*-tests evaluated the participants’ outcomes across different subgroups as well as within subgroups (pre- versus post-participation in Fearless).

For the HAES, the percentage of the day participants spent in each activity level was multiplied by the respective time (minutes) they spent in each of the four time periods. The total daily time spent in each activity level (minutes) was then calculated for every participant using the summation of time spent in that activity level across all four time periods. The daily values were then extrapolated to determine the total weekly time spent in each activity level (e.g., total weekly time inactive = 5 x total daily time inactive during weekday + 2 × total daily time inactive during weekend).

For the IPAQ, the total amount of time (MET-minutes/week) participants spent in each activity level was calculated (MET-minutes/week = MET value for activity level x number of days x total time in activity level).

Sensitivity analyses compared the data from participants who completed only the pre-event questionnaire to those who completed both the pre- and post-event questionnaires to assess for differences and possible biases. Cohen’s d effect size was used to indicate the strength of the association between subgroups. Based upon foundational work from Cohen [42], the effect sizes were defined as either small (≥0.2), medium (≥0.5), or relatively large (≥0.80) and used to inform the interpretation of study outcomes.

Participants’ written feedback was analyzed in accordance with a six-phase framework of reflexive thematic analysis, allowing researchers to inductively code for initial categories [43]. Two researchers (A.C.P. and J.Y.) familiarized themselves with the data and completed separate analyses before coming together to compare preliminary codes [36]. Further discussion and additional data review resolved discrepancies in the preliminary codes and aided in the development of evolving codes. These codes were further collated into major themes, reviewed for distinctiveness and consistency, and continuously refined throughout the data analysis process [43].

## 5. Conclusions

Individuals with CHD face unique internal and external barriers that prevent them from meeting the recommended physical activity guidelines and reaping the associated physical and mental health benefits. Community-based physical activity interventions, such as Fearless, can promote safe and health-appropriate physical activity opportunities for individuals with CHD of all ages. Fearless successfully engaged individuals with CHD who were less active and older, populations that are often challenging to engage and would likely benefit the most from such an intervention. After one Fearless event, 50% of the research participants reported incremental improvements to their physical activity levels. Fearless events provide a framework for sport and recreation leaders who aim to promote physical activity amongst individuals with CHD. Future research should examine the impact of more frequent (i.e., weekly or monthly) Fearless events and evaluate the short- and long-term physical and mental health outcomes of such interventions for this population.

## Figures and Tables

**Table 1:** Age and sex of fearless participants who completed the research questionnaires.

Participant Demographics			Intrinsic Motivation	Perceived Competence Scale	Habitual Activity Estimation Score ^1^	International Physical Activity Questionnaire
Youth	Pre and Post	n = x Age Sex	n = 8 10.25 ± 3.7 5M, 3F	n = 9 10.44 ± 3.5 5M, 4F	n = 7 10.00 ± 3.92 4M, 3F	
	Pre only	n = x Age Sex	n = 8 7.50 ± 3.2 4M, 4F	n = 7 6.85 ± 2.79 4M, 3F	n = 6 7.83 ± 3.31 3M, 3F	
Adult	Pre and Post	n = x Age Sex	n = 7 43.14 ± 11.4 0M, 7F	n = 8 42.00 ± 11.0 0M, 8F		n = 5 40.00 ± 11.24 0M, 5F
	Pre only	n = x Age Sex	n = 9 38.56 ± 8.75 2M, 7F	n = 8 39.13 ± 9.17 2M, 6F		n = 8 38.75 ± 10.48 2M, 6F

^1^ Only complete 7-day participant data for the HAES was included in the table. There were no differences in activity level between those who completed only the weekday or weekend questionnaires and the combined 7-day (both weekend and weekday) responses.

**Table 2:** Participants’ intrinsic motivation and perceived competence as reported by the intrinsic motivation inventory and the perceived competence scale.

Age Group	Pre- and/or Post-Event ^1^	Data Reported	Intrinsic Motivation Inventory	Perceived Competence Scale
Youth	Pre-event all	n = x Mean and SD Sex	n = 8 5.32 ± 1.10 4M; 4F	n = 9 5.47 ± 1.35 5M; 4F
	Post-event all	n = x Mean and SD	n = 8 5.00 ± 0.89	n = 9 5.66 ± 1.27
	Pre- vs. Post-event	*p*-value Cohen’s d	0.19 0.31	0.62 0.14
	Pre-event only	n = x Mean and SD Sex	n = 8 5.71 ± 0.69 4M; 4F	n = 7 6.63 ± 0.33 4M; 3F
	Pre- only vs. Pre-event all	*p*-value Cohen’s d	0.49 0.42	0.03 1.18
Adult	Pre-event all	n = x Mean and SD	n = 7 5.06 ± 0.99	n = 8 4.18 ± 1.93
	Post-event all	n = x Mean and SD	n = 7 5.00 ± 0.81	n = 8 4.43 ± 1.50
	Pre- vs. Post-event	*p*-value Cohen’s d	0.60 0.06	0.33 0.14
	Pre-event only	n = x Mean and SD Sex	n = 9 4.68 ± 1.38 2M; 7F	n = 8 4.78 ± 2.23 0M; 7F
	Pre only vs. Pre-event all	*p*-value Cohen’s d	0.53 0.32	0.42 0.34

^1^ All = all research participants; Only = research participants who only completed the pre-event questionnaires; Pre vs. Post = research participants who completed both pre- and post-event questionnaires.

**Table 3:** Description and content of Fearless events.

	Spring	Summer	Fall	Winter
Fearless Event Schedule (no seasonal changes)	Introduction/Icebreaker Physical Activity #1 Education Session#1 Break—Snack/Rest Physical Activity #2 Education Session #2 Physical Activity #3 ^1^ End of Session Discussion/Feedback			
Physical Activity Opportunities	Yoga Frisbee Bocce Nature Walk Cooperative Games Yoga Frisbee Bocce Nature Walk Cooperative Games ^2^	Tai Chi Zumba Cooperative Games ^2^	Badminton Outdoor Low Ropes Course Cooperative Games ^2^	Capoeira Pickleball (select locations) Cooperative Games ^2^
Educational Sessions	Self-Awareness 24 h Movement Guidelines	Physical Activity Communication Body Awareness	Self-Monitoring Breathing Community Resources for Physical Activity	Creating Connections Goal Setting

^1^ Depending on the length of the activities, some events consisted of only two physical activities. ^2^ Cooperative Games: small recreational activities, requiring teamwork, cooperation, and creativity. Examples include versions of tag, Frisbee games, and team-based obstacle races.

## Data Availability

The data that support the findings of this study are not publicly available, due to privacy and ethical concerns, but can be provided upon request to the corresponding author.

## Supplementary Materials

The following supporting information can be downloaded at: https://www.mdpi.com/article/10.3390/jcdd10010011/s1, Supplementary File S1: Example Lesson Plans for Each Fearless Event, Supplementary File S2a: Post-event Feedback Forms for Adults and Children, Supplementary File S2b: Summary of recommended changes by event age group, Supplementary File S2c: Details of constructive feedback and suggestion comments by event age group.
