# Supplementary material for: Fearless in Physical Activity: The Implications of Community-Based Physical Activity Interventions on Children, Adolescents, and Adults with Congenital Heart Disease
Source: J Cardiovasc Dev Dis. 2022 Dec 28;10(1):11. doi: 10.3390/jcdd10010011 (PMC9861746; doi:10.3390/jcdd10010011)
Supplement: Supplementary file 1 [file jcdd-10-00011-s001.zip › jcdd-2060519-supplementary.pdf]

Journal of
Cardiovascular
Development and Disease

MDPI

Supplementary Files

Supplementary File 1: Example lesson plans for each Fearless event

All Ages Fearless Event Sunday, July 23nd 12-3pm

Time	Activity	Equipment Needed
12:00-12:05 pm	Introduction<br>• Outline the facilitators, CCHA representative, goals for the event (physical literacy through education, activities and movement)<br>• Agenda for the event!	• Have agenda written on the large post it board
12:05-12:15 pm	Icebreaker (Active)<br>• Icebreaker for participants to increase familiarity and comfort with one another.	• Beach Ball
12:15-12:45 pm	Physical Activity #1<br>• Warm-up, yoga movement<br>• Animal Movement Game	•
12:45-1:10 pm	Learning Session #1: Physical Activity & Me<br>• Body Outline Drawing activity – different components of physical literacy & fitness<br>• Self-Monitoring tool – Fire-Breathing Dragon Craft or Mala Beads	• Physical activity and me handout
1:10-1:40 pm	Physical Activity #2<br>• Rae – Lead the group through family oriented physical literacy activity * anything fun & movement based	
1:40-1:55 pm	Break	• Snacks
1:55 -2:20 pm	Education Session #2: Communication/Creative Movement Craft<br>• Minefield, Telephone Charades, Back to Back drawing race<br>• Wind Sock Kite	• handout
2:20-2:45 pm	Physical Activity #3<br>• Carnival Games (indoor gym games)	•
2:45-3:00 pm	Conclusion<br>Wrap up and feedback	• Feedback forms

Fearless Physical Activity Winter Pilot – Youth Session

Time	Activity	Equipment Needed
1:00-1:05 pm	Introduction<br>• Outline the facilitators, CCHA representative, goals for the event (physical literacy through education, activities and movement)<br>• Agenda for the event!	• Have agenda written on the large post it board
1:05-1:15 pm	Icebreaker (Active)<br>• Icebreaker for participants to increase familiarity and comfort with one another.	• Beach Ball
1:15-1:45 pm	Physical Activity #1<br>Yoga (Obstacle Course as a second option)	• Yoga Mats
1:45-2:10 pm	Learning Session #1<br>Winter Tips & Self-Awareness<br>• Inner Traffic Light Activity – Activation to Relaxation relay<br>• Winter Dress up Activity	• Inner Traffic Light handout
2:10-2:40 pm	Physical Activity #2<br>Outdoor – Snowshoeing	• Snowshoes/YMCA Staff to show us around
2:40-2:50 pm	Break	• Snacks
2:50 -3:15 pm	Education Session #2<br>Physical Activity & Me<br>• Charades as an activity to create discussion about the different types of physical activity we can do throughout the day.<br>• GET Creative☺ In groups you will have 5 minutes to come up with a new game (3 rules) and teach the group.	• Charades cards<br>• Need equipment for games
3:15-3:45 pm	Physical Activity #3<br>Capoeira (Martial Arts movement)<br>Option #2 Cooperative games	• Equipment for the cooperative games
3:45-4:00 pm	Conclusion<br>Wrap up and feedback	• Feedback forms printed and "Helpful Tips" Handouts

Physical Activity Session #1

Option 1 **Yoga**
Objective: To introduce participants to the practice and basic poses of yoga
Timeline: 5 minute demonstration, 10 minutes learning poses and modifications, 10 minute yoga practice, 5 minute savasana
Equipment: Yoga mats (optional)
Participants will learn the basic patterns of vinyasa yoga and breathing practices. The series taught will include sun salutation series: mountain pose, forward fold, flat back, plank, chaturanga (knee pushup), upward dog, downward dog, warrior I, warrior II, balancing series: tree pose, airplane pose, boat pose, chair pose, resting series: savasana, child's pose, reclining bound angle.

Option 2* **Obstacle Course** – this is an extra activity that can be added in if we are low on time or if interest is low for yoga.

Learning Session #1

Self-Awareness Lesson: Inner Traffic Light

Activation to relaxation activity; have participants do an activity that gets their heart rate up and have them do an activity that requires concentration. Create discussion around the different levels of activation.

Tips for Outdoor Activity

Tips for dressing for the season; keeping warm and dry☺ (WinterSmart). The importance of layering.
Dress up relay game.

Physical Activity Session #2

Option 1 **Orienteering (outdoor)**
Equipment: maps, markers, compasses
Timeline: 10 minutes to explain compass use and orienteering, 20 minutes to complete all checkpoints
Participants will learn to navigate by foot between checkpoints using a compass and map while racing other individuals to the finish line.

Option 2 **Snowshoeing (outdoor)**
Objective: Participants will learn to snowshoe in local scenic trails. Learning new styles of locomotion challenges coordination and balance while providing a cardiovascular challenge.
Fitness components: coordination, cardiovascular endurance, balance
Equipment: Snowshoes
Timeline: Participants will be given 10 minutes to bundle up, put on snowshoes and familiarize themselves with the movement. 10 minutes will be divided playing the following 2 games. 2 minutes will be spent explaining the game and the game will be played for 8 minutes.

Learning Session #2

Physical Activity & Me

Share physical activity interests with the group. Have everyone tell the group about one activity they already like to do, and one activity they want to try! Ask who else wants to try that activity?

- What are some active things you like to do as a family?
- What are some active things you like to do with friends?
- Brainstorm different types of activities youth like to do (Think outside the box! Remind kids to be creative and include any activities that use any kind of movement)
- Brainstorm a list of *want to do* activities (provide alternatives to turn *can't do* into *can do* activities i.e. providing skill-building skating games or one-on-one hockey instead of playing team hockey)

Charades

Get kids to act out different types of activity and have everybody guess what they are acting out; talk about the different types of activity and when we do them throughout the day.

Get Creative – Split into groups and create a new game or activity that includes different aspects from each different type of physical activity. Teach everyone the rules of the game and we will all play.

Physical Activity Session #3

Option 1 **Capoeira**

Objective: To introduce participants to the basic steps involved in capoeira.
Equipment: Mirror (optional)
Timeline: 5 minute demonstration, 10 minutes learning steps, 10 minutes combinations, 5 minutes partner mirroring
Participants will be introduced to the basic components of the non-contact movement and gymnastics-based martial art. The movements participants will learn are: ginga, balanca, negativa, macaco, rolê.

Option 2 **Cooperational Games (indoor/outdoor)**

Objective: Participants will learn team-building games
Fitness components: balance, flexibility, coordination
Equipment: string, construction paper
Timeline: 10 minutes will be spent playing each game: first 2 minutes will be spent explaining the game, 6 minutes will be spent playing the first version of the game, 4 minutes will be spent playing a variation of the game.

Spider web

Participants begin on one side of a vertical maze of string. Participants are directed to work together to traverse the maze without touching the string. Variation: participant travelling through the maze is blindfolded, participants cannot use their arms

Swamp Crossing

Divide into groups of 3. Give each team 3 "rocks" (construction paper cutouts). Make a start and finish line (maybe 30 feet apart) and tell them that they must get their whole team across without touching the water, using only these rocks to get across. Variations:

Only one foot can be on a rock at a time, only 2 "rocks"

Tic Tac Toe Tag
Objective: To make a line of 3 on the grid with your assigned shape before the other team

Facilitators will make a grid with 9 squares at one end of the room. Participants will be divided into 2 teams, one will be Xs one will be Os. Each team will be given 4 either Xs or Os. The first 4 participants will race walk across the room and place their piece. Once they are all placed, participants will move their pieces around or steal the opposite team's pieces to create a line of 3. Movement style can be changed, or the markers made heavier or an awkward shape for an additional challenge.

Supplementary File 2: Feedback forms and recommendations received

Supplementary File 2a: Feedback forms given to Fearless participants

Fearless Physical Activity

Event Feedback Form

General Feedback:

Have you ever attended a Fearless Event before? Yes ❑ No ❑

	Strongly Disagree		Neutral		Strongly Agree
1. I enjoyed the Fearless Physical Activity event	1	2	3	4	5
2. I will recommend this event to others with CHD	1	2	3	4	5
3. The time for each session was appropriate	1	2	3	4	5
4. The leader was easy to understand	1	2	3	4	5
5. The information and activities were well-organized	1	2	3	4	5
6. I would be interested in attending another Fearless Physical Activity event	1	2	3	4	5

7. Did you find the activities/content relevant for your condition? Yes ❑ No ❑

   If no, please explain why:

   ____

   ____

   ____

Appendix D: Event Feedback Form Version #1 (2016-12-19) *page 1 of 3*

Protocol # and Investigator/Coordinator Initials: #16/106X PL/JY

Session Feedback:

We would really appreciate it if you could rate each session and provide any suggestions or comments that you might have. You can rate the sessions using any number from 1 to 5. A “5” means that you thought the session was perfect. A “1” means that the session was not helpful and really needs to be improved or changed.

	Needs to Change				It was Perfect
**<< Title of Morning Activity Session #1 >>**	1	2	3	4	5
Comments and suggestions:					
**<<Title of Morning Education Session>>**	1	2	3	4	5
Comments and suggestions:					
**<< Title of Morning Activity Session #2 >>**	1	2	3	4	5
Comments and suggestions:					
**<< Lunch Physical Literacy Session>>**	1	2	3	4	5
Comments and suggestions:					
**<<Title of Afternoon Activity Session #1 >>**	1	2	3	4	5
Comments and suggestions:					
**<<Title of Afternoon Education Session>>**	1	2	3	4	5
Comments and suggestions:					
**<<Title of Afternoon Activity Session #2 >>**	1	2	3	4	5
Comments and suggestions:					

Appendix D: Event Feedback Form Version #1 (2016-12-19) *page 2 of 3*

Protocol # and Investigator/Coordinator Initials: #16/106X PL/JY

Comments and Suggestions:

If you have any other comments or suggestions about how we could make this event more helpful, please take the time to share your ideas.

Thank you for coming to our special event.

We really appreciate your time in sharing your thoughts with us!

OPTIONAL

If we have questions or would like to talk with you about your suggestions to improve the Fearless event, could we contact you?

____ No ___ Yes, please contact me using the following information

email:

text message:

phone:

Appendix D: Event Feedback Form Version #1 (2016-12-19) *page 3 of 3*

Protocol # and Investigator/Coordinator Initials: #16/106X PL/JY

Young child feedback form

Fearless Physical Activity Event Feedback

1. Please share something you learned or enjoyed from today's event.

2. How did you feel about the event today? Circle one:

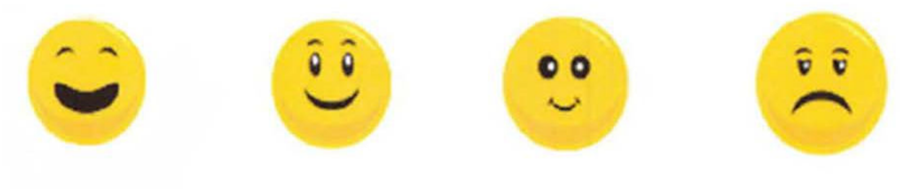

3. How do you feel about attending another event? Circle one:

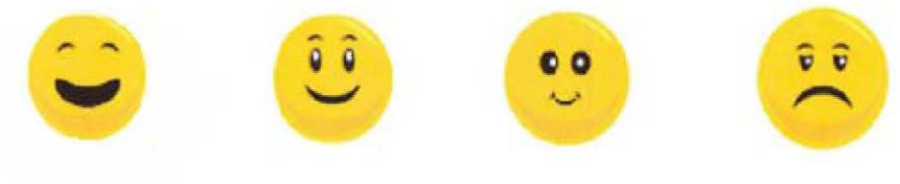

4. Do you have any ideas for the next events? Please write or draw.

		Construct/Suggest[1]			
	Comments[2]	n[3]	Type of feedback	%[4]	Total %[5]
Youth & Children	45	33	4 - Separate age group<br>17- Activity suggestion<br>2 – Have more attendees<br>10 – Event logistics	12%<br>52%<br>6%<br>30%	5%<br>22%<br>2.5%<br>13%
Adults	14	6	2 – Activity suggestion<br>4 – Logistical Suggestion	33%<br>67%	8%<br>16%
All Ages	10	6	1 – Separate age group<br>3 – Have more attendees<br>1 – Logistical suggestion<br>1 – Activity suggestion	16.6%<br>50%<br>16.6%<br>16.6%	4.5%<br>13.6%<br>4.5%<br>4.5%

Supplementary File 2b: Summary of recommended changes by event age group

[1] Construct/Suggest (n) = number of comments that offered constructive feedback or suggestions for change
[2] Comments = total number of comments received
[3] n = number of comments that offered constructive feedback or suggestions for change for each event age group
[4] % = percentage of comments that offered constructive feedback or suggestions for change for each event age group
[5] % of Total Feedback Forms = the percentage of constructive/suggestion comments from among all feedback forms submitted

	**Youth & Children**	**Adults**	**All Ages**
**Activity Suggestions**	*Do more activity (3)*<br>*Yoga*<br>*More variety*<br>*Obstacle course/Relay (2)*<br>*Swimming (2)*<br>*Capture the flag (2)*<br>*Wax Museum (2)*<br>*Core workout*<br>*Tai Chi*<br>*Pilates (2)*	*Pilates*<br>*Rock wall*	*More accessible dance type*
**Logistical Suggestions**	*-Invite doctors to speak*<br>*- shorter education session*<br>*-additional info prior to event (activities, gear needed) (3)*<br>*-different start time*<br>*- have a signal to get everyone's attention*<br>*-exchange contact information*<br>*-improve time management*	*-More promotion to the community*<br>*-Have participants share their condition*<br>*-More discussion for more connection*<br>*- More info prior to event (e.g., itinerary) (2)*	*-Shorten to 2 hours*

Supplementary File 2c: Details of constructive feedback and suggestion comments by event age group

(n) = same comment provided more than one time
